# *Para*-Nonylphenol Impairs Osteogenic Differentiation of Rat Bone Marrow Mesenchymal Stem Cells by Influencing the Osteoblasts Mineralization

**Published:** 2012

**Authors:** Mohammad Husein Abnosi, Malek Soleimani Mehranjani, Mohammad Ali Shariatzadeh, Leila Dehdehi

**Affiliations:** 1 *Biology Department, Faculty of Sciences, University of Arak, Arak, Iran*

**Keywords:** Calcification, Cell differentiation, Mesenchymal stem cell, * Para*-nonylphenol

## Abstract

**Objective(s):**

*Para*-Nonylphenol (*p*-NP) is used in many industries and our previous study showed that *p*-NP causes a reduction in rats bone marrow mesenchymal stem cells (MSCs) viability. The aim of this study was to investigate the effect of *p*-NP on osteogenic differentiation of MSCs.

**Materials and Methods:**

MSCs were isolated and expanded to 3rd passage, then cultured in DMEM supplemented with osteogenic media as well as 0.5 or 2.5 µM of *p*-NP. After 5, 10, 15, and 21 days, the viability and the level of mineralization was determined using MTT assay and alizarin red, respectively. In addition, morphology and nuclear diameter of the cells were studied with the help of fluorescent dye. Furthermore, calcium content and alkalinphosphatase activity were also estimated using commercial kits. Data were statistically analyzed and the *P*<0.05 was taken as the level of significance.

**Results:**

The viability and mineralization of the cells treated with 2.5 µM of *p*-NP reduced significantly after day 10 in comparison with the control group and administration of 0.5 µM. Moreover, chromatin condensation, reduction of nuclei diameter, and cytoplasm shrinkage was observed in the cell treated with 2.5 µM. The calcium concentration and alkalinphosphatase activity of the cells decreased significantly with 2.5 µM of *p*-NP when compared with 0.5 µM and control group.

**Conclusion:**

Adverse effect of *p*-NP was observed on osteogenic differentiation of MSCs at 2.5 µM due to disruption of mineralization. We strongly suggest more investigations on this chemical with respect to other stem cells, especially skin stem cells as *p*-NP is used in the formulation of cosmetics.

## Introduction

*Para*-nonylphenol (*p*-NP) is used in manufacturing a number of commercial products such as latex coating and adhesives, papers and pulps, textile and dyeing, paints, detergents, wetting agents, cosmetics, and pesticides (1). Human is exposed to *p*-NP through food chain, air, water, and various industrial products which are routinely used (1-3). Recent studies have found 56 ng/ml (0.026 µM) and 268 ng/g (1.2 µM) of *p*-NP in human milk and blood plasma, respectively (4). Due to the hydrophobic character of *p*-NP, it can be absorbed and accumulate in the adipose tissue and pose great hazard to the health of human and animals (5-7). Bone marrow which contains both hematopoietic and mesenchymal stem cells is rich in fat (8, 9) thus very much vulnerable to accumulation of this environmental pollutant. Bone marrow mesenchymal stem cells (BMCs) are able to differentiate to osteoblasts, chondrocytes, and adipocyte (10); therefore, it is considered as the main source of bone regeneration and remodeling during its homeostasis (11-13). In animal and cell studies, pollutants such as *p*-NP have been shown to hinder the male sexual development (14), sperm production (15), alter the T cell function (16), induce apoptosis, and inhibit sarcoplasemic/encoplasmic reticulum-type Ca^2+^ pumps (4, 17) after short duration of exposure. In our previous study, we showed that 100 µM of *p*-NP caused a significant reduction of BMCs viability after 36 hr (18), but there was no data available on the effect of *p*-NP on differentiation property of MSCs. Therefore, in this study, we investigated the effects of 0.5 and 2.5 µM of *p*-NP on morphology, viability, calcium concentration, alkaline phosphatase activity, and mineralization of rat bone marrow mesenchymal stem cells following its differentiation to osteoblast. 

## Materials and Methods

***Marrow cell culture***

In the present study, Wistar rats (6-8 weeks old) were purchased from Pasteur Institute (Tehran, Iran) and kept in the animal house of Arak University under standard condition of light and food. The animals were sacrificed by excessive chloroform inhalation and then their tibia as well as femur bones were removed and cleaned from the adherent soft tissue. Then, the two ends of the bones were cut off and bone marrow was flash out using 2 ml DMEM (Dulbecco’s Modified Eagles Medium, Gibco, Germany) supplemented with 15% FBS (Fetal Bovine Serum, Gibco, Germany), 100 U/ml penicillin, and 100 μg/ml streptomycin, (Gibco, Germany). The bone marrow content was centrifuged at 1200 rpm for 5 min and re-suspended in 5 ml DMEM containing 15% FBS and antibiotics then plated in 25-cm^2^ flasks and incubated at 37 °C with atmosphere of 5% CO_2_. Two days after culture initiation, the first medium replacement was performed and then medium was changed two times a week till the bottom of the flask was covered with the cells (till confluency). The cells were trypsinized (trypsin-EDTA, Gibco, Germany) and passed on a new culture flask as the first passage and then the cultures were expanded through two additional subcultures for more purification of the mesenchymal stem cells which were used for further investigation.

***Osteogenic induction***

Mineralization was induced on confluent monolayers of cells with the addition of DMEM containing 15% (v/v) FBS, streptomycin-penicillin, and osteogenic supplements (1 mM sodium glycerophosphate, 50 µg/ml L-ascorbate and 10^-8^ M dexamethasone [all the chemicals were purchased from Sigma- Aldrich company]. Culture flasks were incubated at 37°C with 5% CO_2_ and their medium was changed every 3 days for 21 days (19). 

***Exposure to p-NP***

Stock solution of *p*-NP was prepared in DMSO. The final concentration of DMSO in culture medium was below 0.01%, which at this concentration, DMSO does not affect normal cell growth (5). To perform the assays, cells were cultured in separate culture dishes in presence of DMEM supplemented with osteogenic media for a period of 21 days which represented control and *p*-NP-treated (exposed to 0.5 and 2.5 µM of *p*-NP) groups.

***Cell viability assays***

The viability test on control and treated cells was carried out in an ELISA microplate using 10 µl of MTT (4, 5-dimethylthiazol-2-yl)-2,5-diphenyltetrazolium bromide) solution (5 mg/ml of PBS), where after 4 hr of incubation, the mitochondrial succinate dehydrogenase in the live cells was converted yellow color tetrazolium into violet crystal of formazan. Then, 100 µl of DMSO was added to each well of the plate and formazan crystals were extracted in that following incubation for 30 min in room temperature. The extracted solutions were transferred to another well and absorbance was measured on an automated microplate reader (SCO diagnostic, Germany) at 505 nm. 

***Analysis of morphological changes***

Following *p*-NP treatment in an osteogenic media for 21 days, the nuclear morphology of the cells was studied using Hoechst 33342 at room temperature after 5 min of incubation in the dark. The diameter of the cells was also measured in µM with Motic Image software (Micro optical group company, version 1.2). Hoechst is a fluorescent dye which penetrates the cells through the intact plasma membrane and stain the DNA where the changes in nuclear morphology such as chromatin condensation and fragmentation can be investigated (20). Co-staining the cells with Hoechst and propidium iodide for 5 min at room temperature was used to discriminate between dead and live cells. Propidium iodide is also a fluorescent nucleic acid binding dye which cannot penetrate the membrane of viable cells but readily enter the cell after it loses its membrane integrity (21). The morphology of the cell cytoplasm was investigated using another fluorescent dye (acridine orange) which stains the nuclei green and the cytoplasm orange. The stained cells were washed twice with PBS, examined, and immediately photographed under an inverted fluorescence microscope (Olympus, IX70) equipped with a camera using 40X magnification.

***Detection and quantification of mineralization***

The cells in 6-well plates were washed with PBS and fixed in 10% (v/v) formaldehyde (Sigma-Aldrich) at room temperature for 15 min. The cells were then washed twice in an excess of dH_2_O and 1 mL of 40 mM alizarin red solution (ARS) (pH 4.1) was added to each well. The plates were then incubated at room temperature for 20 min with gentle shaking. After that, the excess of dye was poured off and the plates were washed four times with dH_2_O. Stained cells were investigated under light microscopy using an inverted microscope. To quantify the level of absorbed alizarin red, 800 µl of 10% acetic acid (v/v) was added to each well, and the plate was incubated at room temperature for 30 min with gentle shaking. Then, the loosely attached cells were scraped from the plate with a cell scraper and transferred to a 1.5 ml microcentrifuge tube. After vortexing for 30 sec, the slurry was overlaid with 500 µl mineral oil (Sigma-Aldrich), heated at 85°C for 10 min, and then kept on ice for 5 min. The slurry was then centrifuged at 13500 rpm for 15 min and 500 µl of the supernatant was transferred to a new microcentrifuge tube and 200 µl of 10% ammonium hydroxide (v/v) was added to neutralize the acid. An aliquots of the supernatant (100 µl) was read in triplicate at 405 nm in a microplate reader (SCO diagnostic, Germany) and quantified against standard graph (19).

In order to prepare alizarin red standards graph, working ARS (40 mM) was diluted 20 times with a mixture of 5:2 of 10% acetic acid and 10% ammonium to give a concentration of 2000 µM. Then, using serial dilution, standard solution of 2000 to 31.3 µM was prepared and the absorption was taken at 405 nm using a microplate reader. The concentration of the unknown samples was calculated using linear formula Y=0.179X+0.094 with R^2^=0.997 where Y is the absorbance and X is the concentration (mM) of alizarin red. 

***Alkaline phosphatase activity***

Alkaline phosphatase (ALP) activity of control and treated cells in 6-well dishes was determined by p-nitrophenyl-phosphate (pNPP) hydrolysis method, using the ALP assay kit (Darman Kave, Iran). The cells were washed three time with PBS and homogenized in lysis buffer (0.25 M Tris-HCl, Triton X-100, pH:7.5) (22) and the samples were centrifuged at 12000 RPM for 10 min at 4˚C. The supernatant was kept in -20˚C for the analysis of ALP activity and protein content. The total protein content of each sample was determined according to Bradford, using bovine serum albumin (BSA) as the standard. ALP activity was determined in protein lysate based on equal amount of protein using pNPP as substrate according to the manufacturer’s instruction (Darman Kave, Iran). Absorbance at 410 nm was measured using spectrophotometer (T80+ PG instrument ltd, England) and then ALP activity was determined from a pNPP standard curve.

***Calcium concentration***

Cells in 6-well plates including the control and treated ones were first washed twice with PBS and then their calcium content was extracted in 50 µl of 0.5 N HCl for 24 hr (23). The amount of calcium was determined using commercial kit (Darman Kave, Iran) and the developed color was measured at 575 nm using spectrophotometer (T80+ PG instrument ltd, England). 

***Statistical analysis***

Statistical evaluation of the data was performed using one-way analysis of variance (ANOVA) Tukey’s test, with the help of SPSS. Results were shown as mean±SD and *P*<0.05 was accepted as the minimum level of significance.

## Results

***Effect of p-NP on cell viability***

**Figure 1:**
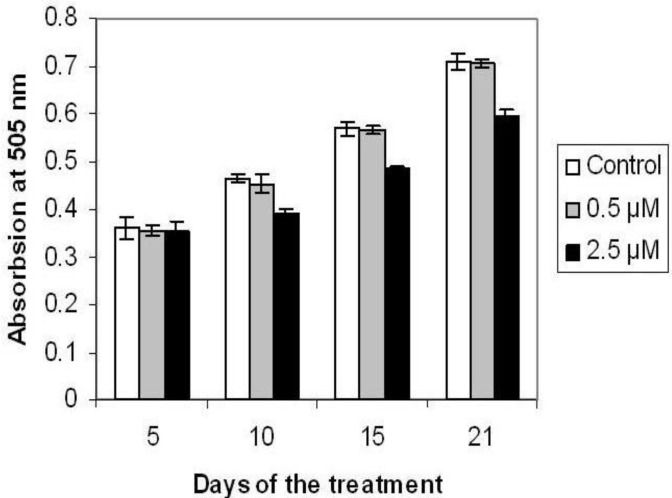
Effect of different concentration of *para*-nonylphenol (*p*-NP) on cell viability in osteogenic culture of BMCs based on MTT assay after 5, 10, 15, and 21 days of treatment. Values are mean±SD (ANOVA, Tukey’s test, *P*<0.05). (*) shows the level of significance at 0.05 and (**) shows the level of significance at the 0.001

Cell viability assay (Figure 1) showed that 2.5 µM of *p*-NP significantly decreased the viability of bone marrow mesenchymal stem cell under osteogenic differentiation on day 10 (*P*<0.05), 15, and 20 (*P*<0.001), but no effect was observed on day 5 as compared with the control. Lower dose of *p*-NP (0.5 cµM) showed no significant effect (*P*>0.05) on the viability of the cells after the treatment periods.

**Figure 2:**
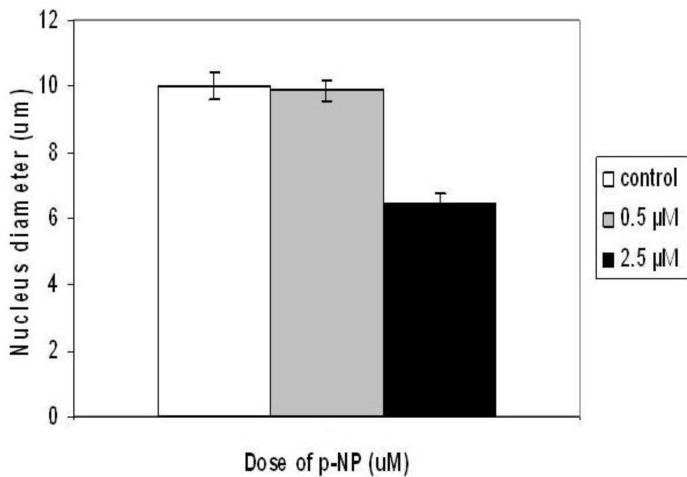
The effect of 0.5 and 2.5 µM *para*-nonylphenol (*p*-NP)on nucleus diameter (µM) of BMCs cultured in osteogenic medium. Values are mean±SD. (ANOVA, Tukey’s test, *P*<0.05). (*) shows the level of significance at 0.001

**Figure 3:**
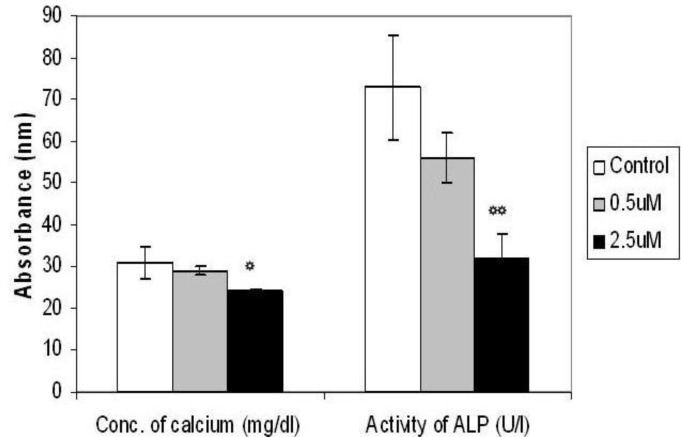
Effect of different concentration of *para*-nonylphenol (*p*-NP)on Ca^2+^ concentration and alkaline phosphatase activity of BMCs cultured in osteogenic medium. Values are mean±SD. (ANOVA, Tukey’s test, *P*<0.05). (*) shows the level of significance at 0.05 and (**) shows the level of significance at the 0.001

**Table 1:** Effect of *para*-nonylphenol (*p*-NP) on mineralization of BMCs cultured in osteogenic medium based on quantitative alizarin red staining

Day Dose	5	10	15	21
0	0.130 ^a^±0.038	0.404 ^a^±0.025	16.871^ a^±0.329	30.242^a^±0.635
0.5 µM	0.147^ a^ ±0.008	0.376 ^a^ ±0.027	16.230 ^a^±0.320	29.534^a^±0.426
2.5 µM	0.121^a^±0.081	0.238 ^b^±0.066	8.931^ b^±0.078	21.527^ b^ ±0.542

Values are mean±SD. Means with the same letter code do not differ significantly from each other (ANOVA, Tukey’s test, *P*<0.05)

***p-NP induced morphological changes of MSCs differentiated cells***

**Figure 4:**
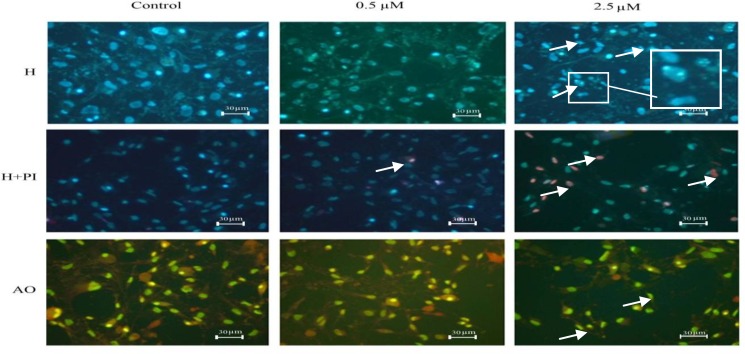
(H) Cells in osteogenic medium stained with Hochest, (Control) Cells in osteogenic media without *para*-nonylphenol (*p*-NP) treatment, (0.5 µM) Cells treated with 0.5 µM of *p*-NP for 21 days, (2.5 µM) Cells treated with 2.5 µM of *p*-NP for 21 days. Nuclear condensation and DNA fragmentation (arrows and enlarged part of the photo) of cells was observed in cells treated with 2.5 µM of *p*-NP. (H+PI) Co-staining of the cells with Hoechst and propidium iodide, (Control) All the cells in control are viable, (0.5 µM) Few of the cells died in 0.5 µM (red neuclei), (2.5 µM) Number of the dead cells increased as compared to the live one when treated with 2.5 µM of *p*-NP for 21 days. (AO) Cytoplasm morphology of cells using acridine orange staining, (Control) Cells in osteogenic media without *p*-NP treatment, (0.5 µM) Cells treated with 0.5 µM, (2.5 µM) Cytoplasm shrinkage was observed in cells treated with o.5 and 2.5 µM of *p*-NP for 21 days (with 40X magnification)

Morphological study of the nuclei of differentiated mesenchymal stem cells treated with 2.5 µM of *p*-NP after 21 day showed significant reduction (*P*<0.001) in nuclei diameter (Figure 2) and chromatin condensation

as well as nuclear breakage (Figure 4-H-2.5 µM). It can be also noticed that *p*-NP at this concentration caused remarkable increase in the death cells (Figure 4-H+PI-2.5 µM) and change in the morphology of cytoplasm (Figure 4-AO-2.5 µM) such as shrinkage and in some cells complete disappearance, as compared with control and 0.5 µM treated cells. 

***Mineralization base on alizarin red staining***

**Figure 5:**
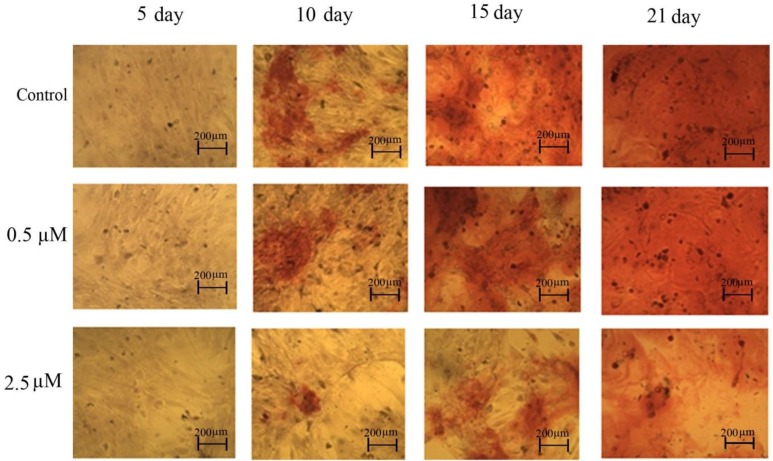
Alizarin red staining for mineral deposition was performed for MSCs after 5, 10, 15, and 21 days of osteogenic induction. (Control) Cells in osteogenic media without *para*-nonylphenol (*p*-NP) treatment, (0.5 µM) Cells in the presence of 0.5 µM of *p*-NP in different treatment period, (2.5 µM) Cells in the presence of 2.5 µM of *p*-NP in different treatment periods (with 10X magnification)

Data showed that the mineralization of cells under osteogenic differentiation is minimum on day 10 and reaches its maximum level on day 21 in control group (Figure 5-control). The treatment of the cells with 2.5 µM of *p*-NP caused significant reduction (*P*< 0.001) in the mineralization from day 10 to day 21 based on quantitative (Table 1) as well as qualitative alizarin red estimation (Figure 5- 2.5 µM) as compared with the control and 0.5 µM treated groups. 

***Calcium concentration and alkaline phosphatase activity***

Calcium concentration of the differentiated cells on day 21 was found to decrease significantly (*P*<0.05) in the group treated with 2.5 µM of *p*-NP as compared with control and 0.5 µM treated groups. In addition, the activity of alkaline phosphatase enzyme was decreased significantly (*P*<0.001) in the cells treated with 2.5 µM of *p*-NP as compared with other groups. However, there was no significant (*P*>0.05) changes between control and 0.5 µM treated groups with respect to intracellular calcium content and alkaline phosphatase activity (Figure 3).

## Discussion

The present study was designed to investigate the effect of *p*-NP on differentiation of MSCs 

to osteoblasts as well as to characterize the cellular and molecular nature of differentiated MSCs in response to this toxicant. Previous studies have shown that *p*-NP enhances apoptosis in cell types such as thymocyte (16), PC12 cells (24), spermatogenic cells, and sertoli cells (25, 26). In this study, the viability of differentiated MSCs in response to 2.5 µM of *p*-NP was reduced significantly on day 10 onwards but no effect was observed on day 5. Lower dose (0.5 µM) of *p*-NP showed no significant effect on viability, therefore we may say that there is dose and time limitation for *p*-NP toxic effect. Since bone matrix is in direct contact with peripheral blood, based on this study, the presence of low dose up to 0.5 µM might be of no harm. However, *p*-NP has hydrophobic character and can accumulate in the adipose tissue (5, 6), so its concentration in the needed time might rise above the harmful dose limitation to show the toxic effect*.* In today’s life,* p*-NP is used in many commercial product such as cosmetics, so continuous exposure to this chemical might be a matter of investigation and public health concern. 

We also found that 2.5 µM of *p*-NP after 21 days of treatment caused chromatin condensation and nuclear breakage as well as cytoplasm shrinkage which all together might be considered as a sign of apoptosis (27) and a reason for significant viability reduction. Many investigators have shown that the *p*-NP causes activation of caspases through internal and external pathways (5, 16, 24, 28, 29) thus the viability reduction of MSCs under osteogenic differentiation might be due to apoptosis. Moreover, *p*-NP induces free radicals of oxygen (30) where this might be another reason of nuclear breakage. In addition, differentiation of MSCs to osteoblasts is followed by changes in cytoskeleton content such as actin (31), where it is well documented that *p*-NP interferes with polymerization of cytoskeleton (7), which can be another reason for cytoplasm shrinkage. 

Our finding showed that the level of mineralization in terms of quantitative alizarin red, calcium concentration, and alkaline phosphatase activity reduced significantly (*P*<0.05) from day 10 in the 2.5 µM group as compared with the control and 0.5 µM treated groups. After a certain period of time, *in vitro* osteogenic mineralization starts (19) with respect to the alkaline phosphatase activity and release of phosphate ion which brings about large influx of calcium ion into the cells (32). The influx of calcium is a necessary step in formation of hydroxyapatite crystal (33) which is the prompt step of bone formation. At this point, with respect to viability and mineralization data, it may be concluded that the effect of the *p*-NP starts as the osteogenic changes occurred in the cell somehow after day 5. 

Investigations have shown that the *p*-NP acts as a xenoestrogen which mimics estrogen action (1, 4, 34) and on the other side MSCs express estrogen receptors (ERs) (35). Dexamethasone is one of the osteogenic supplements and acts as estrogen antagonist (36, 37) therefore binding of* p*-NP to ERs might be one of the reason for reduction of osteogenic processes. Dexamethasone down-regulates calcification-inhibitor molecules gene (38) which causes acceleration of osteogenic differentiation (39) where similar to *p*-NP, might up-regulate the gene due to structural similarity with dexamethasone (1, 40). This probable mechanism needs to be clarified and further investigations are required.

As mentioned earlier, some studies have shown that *p*-NP induces oxidative stress and increases the level of reactive oxygen species in human blood neutrophils (31, 36). The oxidative stress induced by oxygen free radicals inhibits osteogenic differentiation processes (41) thus it might be another reason why *p*-NP caused impairment in osteogenic differentiation processes. Furthermore, osteogenic differentiation depends on Wnt signaling (42, 43), where in this pathway, in the presence of the β-catenin and ICF/LEF factor, the activation of alkaline phosphatase genes takes place. One study has shown that oxygen free radical can inhibit expression of alkaline phosphatase gene by disrupting Wnt signaling (41) which might be a reason for significant reduction of enzyme activity in this study. In addition, investigators showed that free radicals can cause inhibition of calcium channel and disruption of calcium homeostasis (21, 44, 45) which itself might be a reason for significant reduction of calcium influx due to *p*-NP toxicity. 

## Conclusions

Altogether, it is to be mentioned that the MSCs are pluripotent stem cells that can differentiate into osteoblast and are also considered to be a major source of bone formation and remodeling (13, 46) thus their health should be under a great consideration and attention. As in industrial area, the concentration of *p*-NP in some human samples (4, 47) is approaching the harmful range, therefore it might have profound effect on bone homeostasis and remodeling. These findings not only makes it necessary to pay attention to the health of the bone tissue but also other adult stem cells such as skin stem cells which might come in close contact with *p*-NP, when used as an emulsifying agent in formulation of cosmetics. 
